# Quantitative Risk Stratification of Oral Leukoplakia with Exfoliative Cytology

**DOI:** 10.1371/journal.pone.0126760

**Published:** 2015-05-15

**Authors:** Yao Liu, Jianying Li, Xiaoyong Liu, Xudong Liu, Waqaar Khawar, Xinyan Zhang, Fan Wang, Xiaoxin Chen, Zheng Sun

**Affiliations:** 1 Department of Oral Medicine, Beijing Stomatological Hospital, Capital Medical University, Beijing, China; 2 Euclados Bioinformatics Solutions, Cary, NC, United States of America; 3 Ongwandada Resource Center, Queen’s University, 191 Portsmouth Avenue, Kingston, ON, Canada; 4 Ross University School of Medicine, 2300 SW 145th Avenue, Miramar, FL, United States of America; 5 Beijing Institute of Dental Research, School of Stomatology, Capital Medical University, Beijing, China; 6 Xiangyang Central Hospital, Affiliated Hospital of Hubei University of Arts and Science, Xiangyang, Hubei Province, China; 7 Cancer Research Program, Julius L. Chambers Biomedical Biotechnology Research Institute, North Carolina Central University, Durham, NC, United States of America; Duke Cancer Institute, UNITED STATES

## Abstract

Exfoliative cytology has been widely used for early diagnosis of oral squamous cell carcinoma (OSCC). Test outcome is reported as “negative”, “atypical” (defined as abnormal epithelial changes of uncertain diagnostic significance), and “positive” (defined as definitive cellular evidence of epithelial dysplasia or carcinoma). The major challenge is how to properly manage the “atypical” patients in order to diagnose OSCC early and prevent OSCC. In this study, we collected exfoliative cytology data, histopathology data, and clinical data of normal subjects (n=102), oral leukoplakia (OLK) patients (n=82), and OSCC patients (n=93), and developed a data analysis procedure for quantitative risk stratification of OLK patients. This procedure involving a step called expert-guided data transformation and reconstruction (EdTAR) which allows automatic data processing and reconstruction and reveals informative signals for subsequent risk stratification. Modern machine learning techniques were utilized to build statistical prediction models on the reconstructed data. Among the several models tested using resampling methods for parameter pruning and performance evaluation, Support Vector Machine (SVM) was found to be optimal with a high sensitivity (median>0.98) and specificity (median>0.99). With the SVM model, we constructed an oral cancer risk index (OCRI) which may potentially guide clinical follow-up of OLK patients. One OLK patient with an initial OCRI of 0.88 developed OSCC after 40 months of follow-up. In conclusion, we have developed a statistical method for qualitative risk stratification of OLK patients. This method may potentially improve cost-effectiveness of clinical follow-up of OLK patients, and help design clinical chemoprevention trial for high-risk populations.

## Introduction

Oral cancer is one of the major public health problems worldwide, as well as a major cause of cancer morbidity and mortality [1, 2]. In the United States, approximately 28,030 new cases are estimated and 5,850 cases are estimated to die in 2014 [1]. In China, the overall rates of incidence and mortality for oral cancer were 2.93 and 1.26 per 100,000 persons in 2011, and the age-standardized rate of incidence was 2.22 per 100,000 persons [3]. Oral squamous cell carcinoma (OSCC) is the most common type of oral cancer, which usually develops from precancerous lesions such as oral leukoplakia (OLK) and erythroplakia, and histopathologically follows a step-wise pattern of hyperplasia, dysplasia and squamous cell carcinoma [4, 5]. Overall survival of OSCC patients remained unchanged despite the advances in radiotherapy and chemotherapy [1]. The five-year survival rate for patients with early and localized lesions is ~80%, whereas it is only 19% for patients with distant metastasis [6]. Thus it is important to assess precancerous lesions and diagnose OSCC early.

OLK is defined as “a white plaque of questionable risk having excluded other known diseases or disorders that carry no increased risk for cancer” [7, 8]. And the annual age-adjusted incidence rates of OLK varied from 1.1 to 2.4 in male and from 0.2 to 1.3 in female per 1,000 persons in India, and the prevalence varied from 0.2 to 4.9% [9]. In Japan, the age-adjusted incidence rate was 4.1 in male and 0.7 in female per 1,000 person-years [10]. Histopathologically, OLK presents as hyperkeratosis of the squamous epithelium in oral cavity. Months or years are needed for hyperkeratosis progress to cancer. The overall chance of malignant transformation is 3.6% [11] and can be up to 12.9% in some populations [12–14]. This situation creates a huge burden on health care and therefore, there is a need of risk stratification for OLK patients to improve the cost-effectiveness of clinical follow-up.

OLK lesions with a red component, ulceration, or certain topography (granular, nodular, or verrucous) are more likely to develop malignancy [8, 15]. Being subjective in nature, visual inspection depends on clinical experience of the physician, and mucosal appearance of early-stage cancer may appear benign [15]. Histopathology remains the golden standard and the presence of dysplasia often indicates a high risk of cancer [16]. Unfortunately this invasive approach cannot be repeated during follow-up due to poor acceptance by patients. Diagnosis of dysplasia is also subject to experience of the pathologist and sometimes consensus among pathologists is poor [17].

Several other measures are available for clinicians to assess OLK lesions: 1) Visual assessment of the physico-chemical properties, such as toluidine blue staining [18], fluorescence spectroscopy [19]: These methods are easy and quick to use, yet less specific [15, 18, 20]. 2) Laboratory assessment of cellular markers: Exfoliative cytology in conjunction with DNA quantitative analysis [21], micronucleus analysis [22] and nucleolar organizer regions [23], has already been used routinely for diagnosis of OSCC [24]. Its sensitivity and specificity has been reported up to 100% [6, 21, 25–27]. However, some other studies have shown that exfoliative cytology is of no value in detecting mucosal changes that are not readily visible to the naked eyes [18]. Although qualitative assessment (“negative for OSCC”, “positive for OSCC”, or “atypical lesion”) works well for OSCC diagnosis, this method has limited use in assessing cancer risk of those negative and atypical cases. 3) Laboratory assessment of molecular markers: Chromosome *in situ* hybridization, immunohistochemistry, real-time PCR, gene microarray and proteomics have been used for detection of alterations in DNA, mRNA and protein [28]. Although these molecular tools have shown promising results with improved accuracy of cancer diagnosis, they are usually expensive and require high-quality biopsy samples.

In this study, we developed a statistical model for quantitative risk stratification of OLK. A risk index metrics was established to reflect the probability of OSCC. Our main purpose is to distinguish high-risk OLK from low-risk OLK based on data collected by exfoliative cytology, and therefore to potentially improve cost-effectiveness of clinical follow-up.

## Materials and Methods

### Clinical subjects, clinical data and follow-up

We recruited and followed patients from March 2008 to October 2014. The mean, maximum and minimum follow-up time for the OLK patients was 46 months, 74 months and 20 months respectively. During follow-up, changes in clinical signs and symptoms of all subjects were documented through clinical examination and phone calls. Malignant transformation was confirmed by histopathology.

Exfoliated cells were collected from oral mucosa of patients with OLK (n = 82), OSCC (n = 93), and healthy subjects (n = 102) in the outpatient clinic (Table 1). Those who smoked more than 1 pack year were defined as smokers, and a pack year is defined as twenty cigarettes smoked every day for one year. Those who drunk more than 14 g alcohol per day for one year were regarded as drinkers. A ‘standard drink’ is equivalent 14 g alcohol per day [29]. OSCC was classified according to the TNM classification: Stage 0: Tis (carcinoma in situ), N0 (no regional lymph node metastasis) and M0 (No distant metastasis); Stage I: T1 (tumor 2 cm or less in greatest dimension), N0 and M0; Stage II: T2 (tumor more than 2 cm but no more than 4 cm in greatest dimension), N0 and M0; Stage III: T1/T2, N1 (metastasis in a single ipsilateral lymph node, 3 cm or less in greatest dimension) and M0, or T3 (tumor more than 4 cm in greatest dimension), N0/N1 and M0; Stage IV: T4 (tumor invades through cortical bone, into deep/muscle of tongue, maxillary sinus, or skin of face; tumor invades masticator space, pterygoid plates, or skull base, or encases internal carotid artery), N2 (metastasis in a single ipsilateral lymph node, more than 3 cm but not more than 6 cm; metastasis in multiple ipsilateral lymph nodes, none more than 6 cm in greatest dimension; metastasis in bilateral or contralateral lymph nodes, none more than 6 cm in greatest dimension)/N3 (metastasis in a lymph node more than 6 cm in greatest dimension), or M1 (distant metastasis). The early stage Stage 0, I and II are defined as the early stage, while the advanced stage is defined as Stage III or IV [30, 31]. In this study, 9.7%, 25.8%, 31.2%, 16.1% and 17.2% cases of OSCC are at Stage 0, I, II, III and IV, respectively, and 24.7% have lymph node metastasis.

**Table 1:** General characteristics of normal subjects, OLK patients and OSCC patients.

	Normal (n = 102)	OLK (n = 82)	OSCC (n = 93)
Age (yr)			
Mean ± SD	44.00 ± 16.00	58.16 ± 11.48	61.70 ± 11.11
Range	22–80	25–85	21–83
Gender			
Male (%)	46 (45.1)	37 (45.1)	45 (48.4)
Female (%)	56 (54.9)	45 (54.9)	48 (51.6)
Site			
Tongue (%)	28 (27.5)	22 (26.8)	41 (44.1)
Gingival (%)	15 (14.7)	33 (40.2)	27 (29.0)
Other (%)	59 (57,8)	27 (32.9)	25 (26.9)
Smoking			
Yes (%)	32 (31.4)	29 (35.4)	31 (33.3)
No (%)	70 (68.6)	53 (64.6)	62 (66.7)
Drinking			
Yes (%)	17 (16.7)	16 (19.5)	22 (23.7)
No (%)	85 (83.3)	66 (80.5)	71 (76.3)

### Ethics Statement

This study was approved by the ethical committee of the Beijing Stomatological Hospital, Capital Medical University, and all patients signed the informed consent before the study.

### Exfoliative cytology

**Fig 1:**
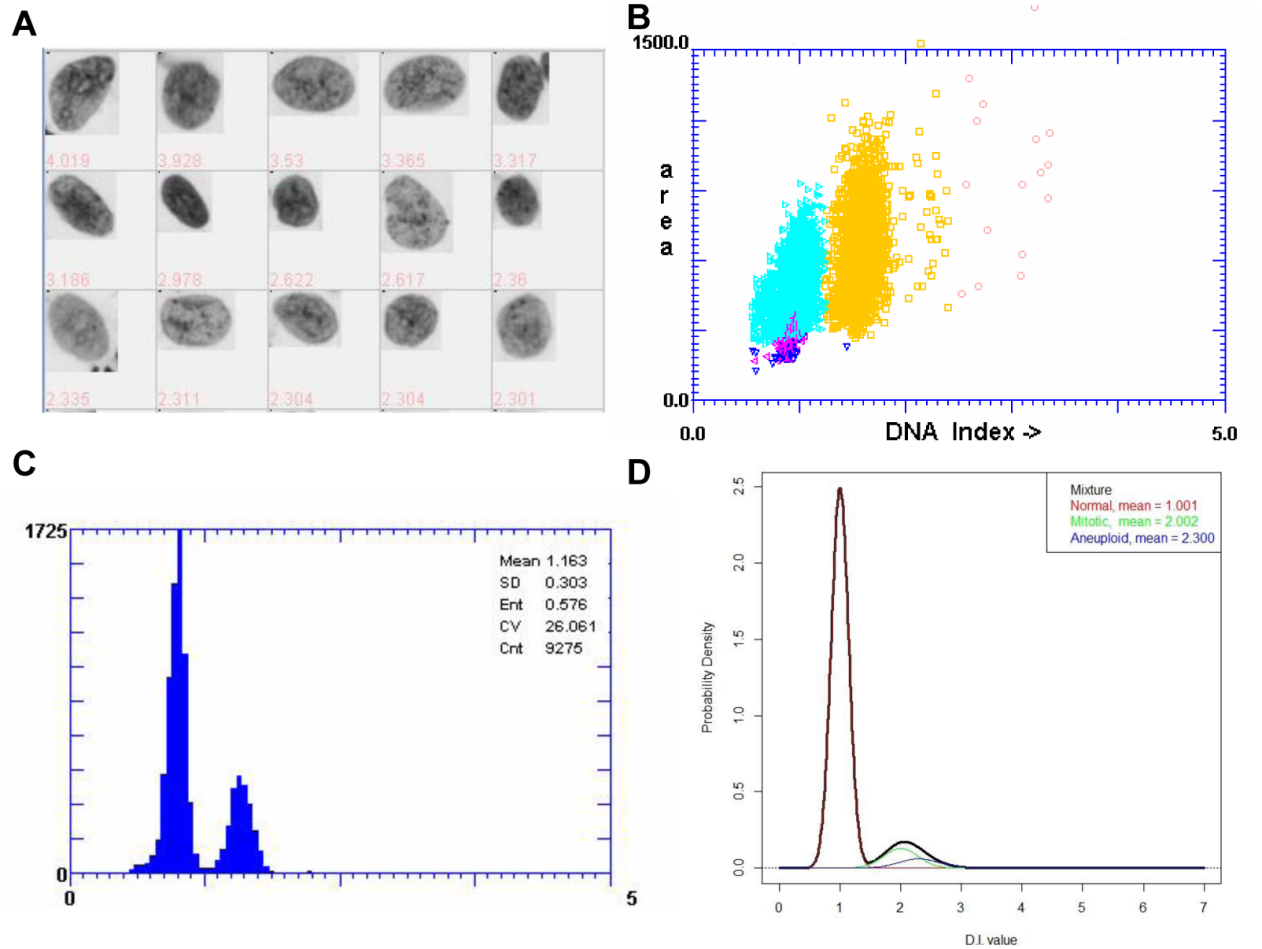
Distribution of DNA contents in exfoliative cytology. (A) Selected cells with abnormally high DI values (>2.3). (B) A scatter plot with y-axis as the area of nucleus and x-axis as DI value. (C) Distribution histogram of DI values of all nuclei. (D) Distribution histogram of DI values of the three cell populations after simulation from normal distribution, diploid cell population (red; μ = 1.001, σ = 0.19), tetraploid cell population (green; μ = 2.002, σ = 0.25) and aneuploidy cell population (blue; μ = 2.300, σ = 0.5). When these three cell populations are merged at the ratio of 0.893:0.092:0.005, a composite distribution histogram (black) can be generated.

Exfoliative cells were collected by using Cervibrush (Motic, China) and stored in a fixative (Motic, China) before Feulgen staining. Cells were smeared onto a dry glass slide and treated with Bohm-Sprenger solution (80ml methanol, 15ml formaldehyde and 5ml acetic acid) for 50 min at 25C, 5N hydrochloric acid for 60min and Feulgen solution for 75min. After wash, the smears were dehydrated using graded ethanol and xylene. DNA-image cytometry and CLASSIFY software (Motic, China) were used for measurement of the DNA index (DI) and other cytologic parameters (132 in total). Twenty of these parameters were regarded useful, such as DI, DNA amount, intensity, radius and area (S1 Table). In this study, we only use the DI value, as show in Fig 1. Rat liver cell nuclear imprint (Motic, China) was used for standardization of DNA image cytometry [32]: integrated optical density (IOD) of diploid cells was between 108 and 122; the ratio of IOD of tetraploid and diploid cells was between 1.9 and 2.1; and the coefficient of variation was below 5%. According to the diagnostic criteria set by the British Columbia Cancer Agency, an aneuploid cell was defined as DI ≥2.3 [33]. A case was defined as “positive (for dysplasia or OSCC)” if there were more than 5 aneuploid cells. Scatter plots and distribution histograms can be generated by the software to reflect the overall status of exfoliated cells (Fig 1B and 1C). A case would be defined as “atypical” if the number of aneuploid cells was between 1 and 5, or “negative” if there was no aneuploid cell.

### Histopathology

For OLK and OSCC, a resection biopsy was taken immediately from the same area under local anesthesia after brush biopsy. Tissues were fixed with buffered formalin and processed for clinical histopathology. Paraffin tissue sections were evaluated by our pathologist according to the standard criteria of the WHO Classification System of Head and Neck Tumors [33]. The features used for diagnosing dysplasia contains: irregular epithelial stratification, loss of polarity of basal cells, drop-shaped rete ridges, increased number of mitotic figures, abnormally superficial mitosis, premature keratinization in single cells, keratin pearls within rete pegs. Mild, moderate, or severe dysplasia is defined if general architectural disturbance is limited to the lower third of the epithelium, extending into the middle third of the epithelium, or greater than two thirds of the epithelium, respectively [34].

### Expert-guided data transformation and reconstruction (EdTAR)

**Fig 2:**
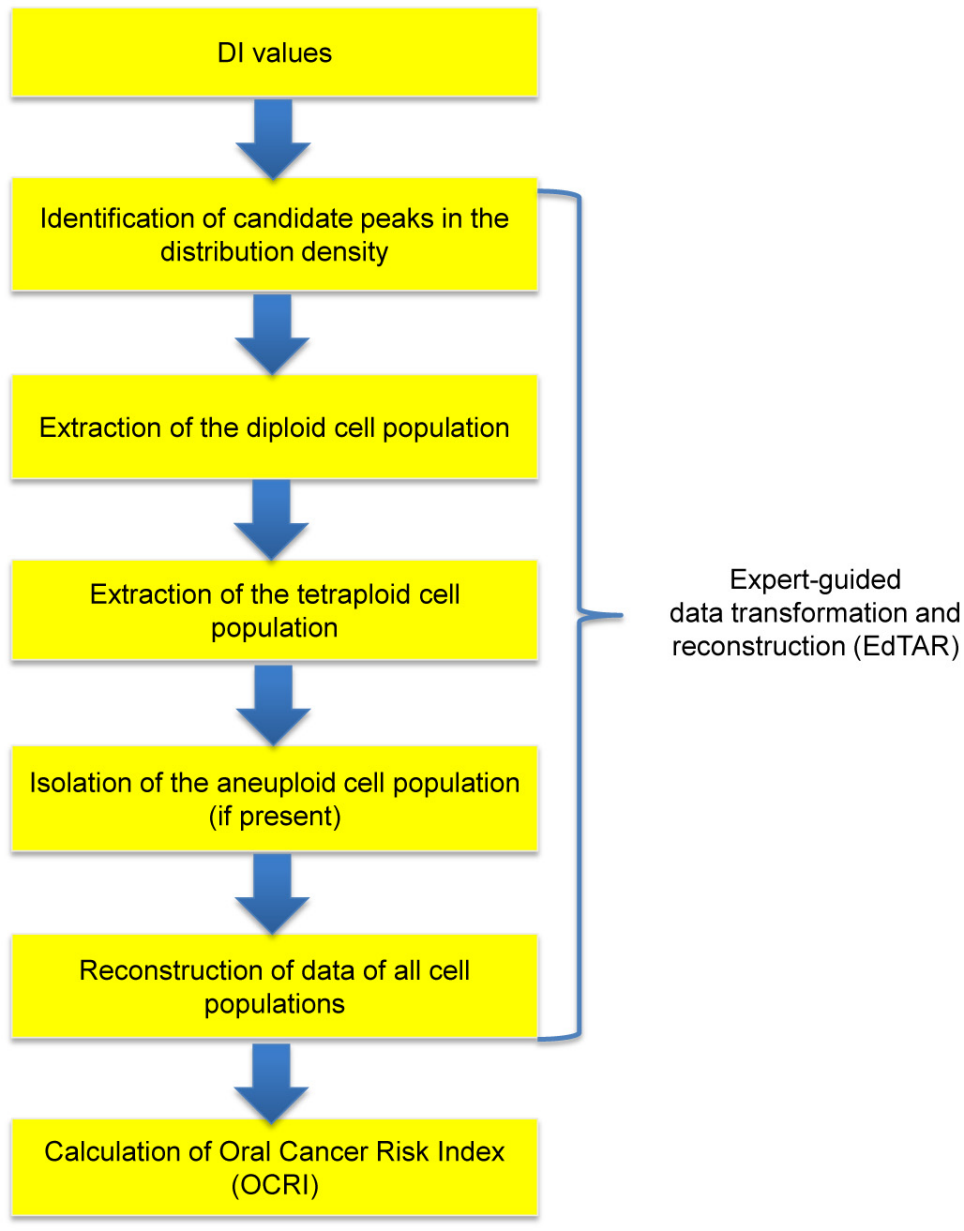
Work flow of expert-guided data transformation and reconstruction (EdTAR). Starting with DI values as the raw data, EdTAR first identified candidate peaks of cell populations. Diploid cell population was extracted and further filtered if more than one population is detected. The same procedure was applied to extract the tetraploid cell population and thus the aneuploid cell population was isolated. Data of these three cell populations were reconstructed across a wide rage [0–8] using the discrete density at each interval. The newly constructed data was used for training the statistical model and calculation of the Oral Cancer Risk Index (OCRI).

In this proof-of-concept study, we only used DI for statistical analysis. EdTAR (Fig 2) was made up of four parts, peak identification, extraction of diploid/tetraploid and isolation of aneuploid, signal amplification, and data reconstruction. Parameter estimation, signal amplification and data reconstruction were carried out with R [35].

### Peak identification

We first aimed to differentiate three possible cell populations, diploid, tetraploid and aneuploidy. We defined the thresholds for peaks that represented each cell population, diploid [0.8, 1.2], tetraploid [1.5, 2.2] and hypertetraploid/aneuploidy [>2.3]. The DI values obtained from the software normally represent a mixture of cell populations and can be displayed in a histogram (Fig 1C). In order to estimate the parameters for each individual cell population, we adopted the procedure of kernel density estimation with the kernel density estimator,

$${\hat{f}}_{h}(x)=n^{-1}\sum_{i=1}^{n}K_{h}(x-X_{i})$$ Equation 1

where $K_{h}(.)=\left(\frac{1}{h}\right)K(\frac{.}{h})$ represents a “kernel function” K and a “bandwidth” *h*. We assumed that DI values were independently selected from a background distribution. To smoothen the histogram, we chose Gaussian distribution as the kernel [36], and finalized on the bandwidth to minimize the mean integrated squared error (MISE), as

$$MISE(h)=E\int {\left({\hat{f}}_{h}-f\right)}^{2}$$ Equation 2

When two or more populations were observed with a fairly large proportion of overlap, bimodal or multi-normal based assumption was made. For such cases, a reflection point was identified if a change in the first derivative sign was observed.

### Extraction of diploid/tetraploid peaks and isolation of aneuploid peak

One key component in our approach was to extract non-informative cell populations, i.e. diploid/tetraploid cell population. To do so, we proposed the sequential steps in the following pseudo code.

1. With the parameter set θ^dt^ for data transformation
2. Candidate peaks obtained on the density distribution from the empirical DI values were stored
3. **for** each i = 1..n peaks **do**
  - Estimate the sample statistics from the left part of the peak
  - Estimate the right part of the distribution
  - Filter out the candidate i^th^ family and retain the mean, SD and count
  - Check the next available peak, if any, against threshold
  - Go back to 3.1 if the peak is < upper bound
  **end**
4. Summarize for candidate cell populations representing the diploid, tetraploid and aneuploid, and store the summary statistics of each population
  - The total number of DI values
  - Mean and standard deviation
  - The number and locations of peaks

### Signal amplification

Our main goal was to quantify the risk via sufficient stratification, which relied on amplifying the informative signals. First, we defined ratios of these three populations as R_1_, R_2_, and R_3_ respectively, with the constraint that

$$R_{1}+\;R_{2}+\;R_{3}=\;1$$ Equation 3

If all three cell populations were detected and their peaks were retained, we achieved the amplified signal of aneuploid population by redistributing the ratio among R_1_, R_2_, and R_3_. The original ratio between two populations (R_1_ and R_2_) were retained and was together weighed as 0.9. If only diploid and tetraploid populations were detected, the original ratio between two populations (R_1_ and R_2_) was retained and together weighed as 0.995, and the hypothetical aneuploid population was sampled from a normal distribution Norm (2.3, 0.3). If a single diploid population was detected, R_1_ will be sampled from a uniform distribution ~Unif [0.75, 0.8], and kept R_1_ + R_2_ = 0.995 and R_3_ [1-R_1_-R_2_]. The hypothetical tetraploid population was sampled from a normal distribution ~ Norm (2.0, 0.3) and the hypothetical aneuploid population from ~ Norm (2.3, 0.3).

### Data reconstruction

For data reconstruction, new variables were created to represent the discrete interval ranging between 0 and 8 (DI values) with 0.5 increments. For each interval, the density estimated from the actual data was used. If any interval is missing, 0.0001 was used as the filler. The procedure is shown in pseudo code format as follows:

1. With the parameter set θ^dr^ for data reconstruction
2. **If** only diploid population exists **do**
  Sample the three population ratios
  Integrate mixture of three theoretical families
  Create densities for all 16 discrete intervals
3. **Else if** both diploid and tetraploid populations exist **do**
**If** tetraploid population mean and standard deviation exist **next**
**Else do**
    Sample the tetraploid population mean and standard deviation
    Sample the aneuploidy population
  Compute the ratio between diploid and tetraploid populations, and sample the ratios of three populations
  Integrate mixture of three theoretical families
  Create densities for all 16 discrete intervals
4. **Else** all three populations exist **do**
**If** the maximum DI value of aneuploidy cells > 8, set it as 8
**If** tetraploid population mean and standard deviation exist **next**
**Else do**
    Sample the tetraploid population mean and standard deviation
  Compute the ratio between diploid and tetraploid populations
  Finalize ratios for all three populations
  Integrate mixture of three theoretical families
  Create densities for all 16 discrete intervals

### Statistical models and performance evaluation

Statistical modeling, variable selection and performance evaluation were done with R [35] and caret package (http://caret.r-forge.r-project.org/). Datasets of “normal subjects” (n = 102) and “OSCC patients” (n = 93) were used to build the prediction models. First of all, we randomly separated the dataset into two parts with 70% samples for model selection and optimization and 30% for testing and evaluation. We selected six statistical models and evaluated their performance, Support Vector Machine (SVM), Random Forest (RRF), Penalized Logistic Regression (PLR), Neural Network (NNET), K-nearest neighbor (KNN), and Classification and Regression Training (CART). To evaluate each model’s performance, we started with the default parameters and further optimized the hyperparameters to achieve the best performance. Using a sampling process, this included ten-fold cross-validation within each pass and repetition for five times. To ensure objective evaluation, we implemented the same random data parsing procedures for internal cross-validation by setting the same seed for any random number generation [37]. These models were ranked according to the area under receiver operating characteristic (ROC), sensitivity and specificity. Based on the performance evaluation, the SVM model was chosen for the following calculation.

### Calculation of the Oral Cancer Risk Index (OCRI)

With the finalized set of EdTAR parameters, the exfoliative cytology data was processed and further used in building the SVM model with a radial kernel function using R kernlab [38] package. To optimize the hyperparameters, we used two-class samples (normal and OSCC) and the same random sampling procedure to recreate the training dataset and test dataset. The training dataset was processed with median centering and column scaling. For the best outcome, we used leave-on-out cross validation and evaluated the model performance on the nine grid cost parameter between 2^(-2)^–64. The final model had a cost of 32 and a hyperparameter sigma of 0.6456. OCRI was calculated as the probability of OSCC for an unknown sample. It ranges between 0 and 1, where 0 indicates the lowest risk of OSCC, and 1 the highest risk of OSCC.

## Results

Clinical data of normal, OLK and OSCC subjects including age, sex, site involvement, smoking and drinking habits are provided in Table 1. According to the original cytology, normal samples from 102 healthy donors were all “normal”. Among 82 OLK samples, 4 were “positive”, 30 “atypical” and 48 “negative”. Among 93 OSCC samples, 89 were detected as “positive”. One example of OSCC is shown in Fig 1A–1C.

### Data transformation and reconstruction by EdTAR

The DI values represented a mixture of cell populations, diploid, tetraploid and aneuploid, and were displayed in a histogram (Fig 1C). In this example, the ratio of the three populations was roughly 0.893:0.092:0.005. We simulated these three populations from three normal distributions, diploid cell population (red; μ = 1.001, σ = 0.19), tetraploid cell population (green; μ = 2.002, σ = 0.25), and aneuploid cell population (blue; μ = 2.300, σ = 0.5). The black curve showed a composite distribution histogram of these three simulated populations at the above mentioned ratio (Fig 1D).

**Fig 3:**
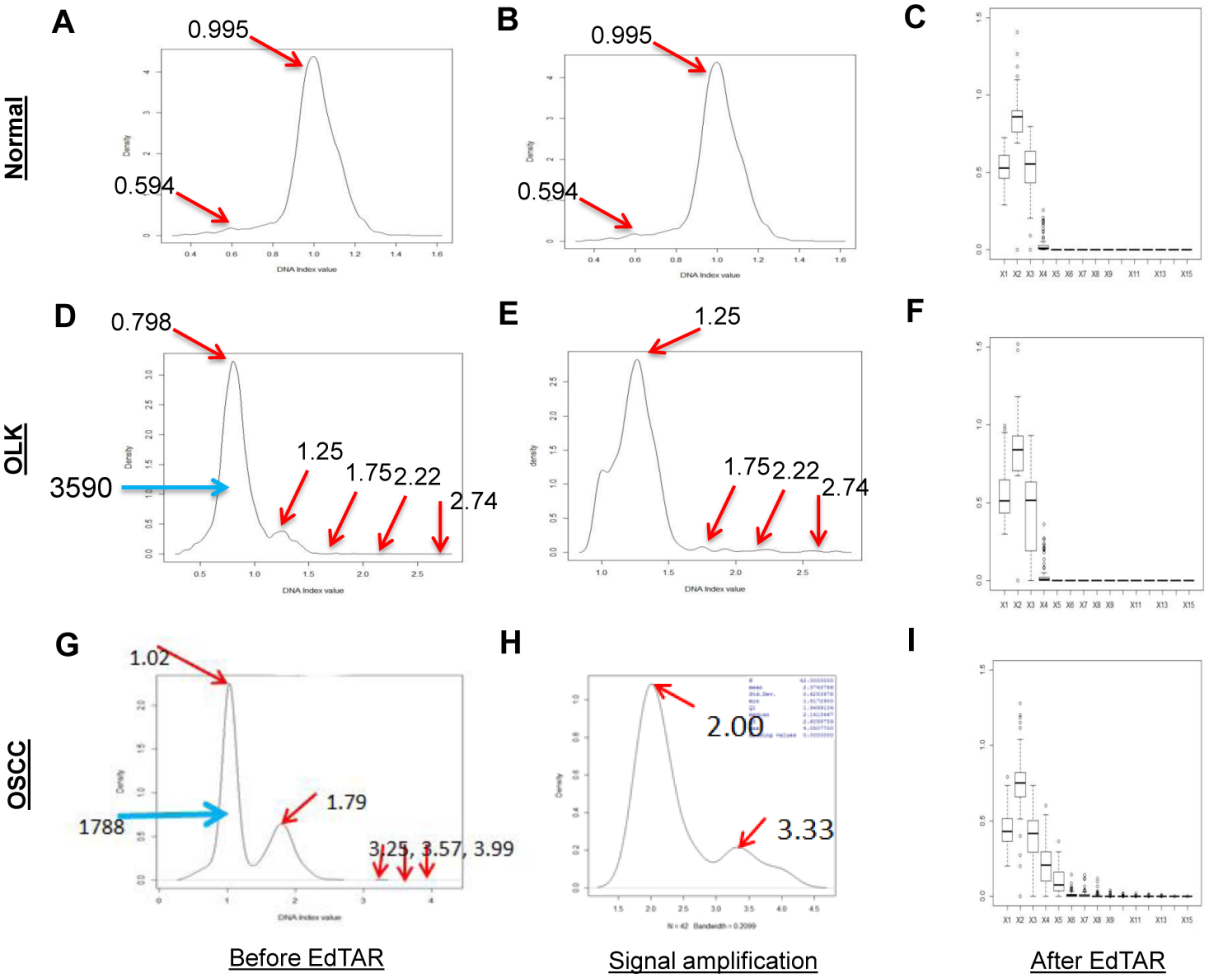
Application of EdTAR in processing data of three samples with pathological diagnosis of normal (A-C), OLK (D-F), and OSCC (G-I). All density plots have x-axis as DI value and y-axis as density. Panel A, D and G showed density plots before data processing by EdTAR. In Panel A, a major peek with a DI of 0.995 represents the diploid cell population, where another small peaks (DI = 0.594) was a minor population possibly due to image processing. In Panel D, a major peek with a DI of 0.798 represents the diploid cell population (3,590 cells). Other than this peak, four peaks with DI values of 1.25, 1.75, 2.22, and 2.74, were present. In Panel G, a major peek with a DI of 1.02 represents the diploid cell population, and a second peak with a DI of 1.79 represents the tetraploid cell population. Other than these two peaks, three peaks with DI values of 3.25, 3.57, and 3.99 were present, and were believed to represent the aneuploidy cell population. Panel B, E and H corresponding with Panel A, D and G respectively were three plots showing the net results of data processing by EdTAR. Signals of the aneuploidy cell populations were amplified in Panel E and H. Panel C, F and I showed boxplots of newly constructed variables after data processing with EdTAR. The x-axis indicated the new variables along a range of DI [0–8] and y-axis the boxplot of available values for each variable.

The first attempt in EdTAR was to identify the peaks. A typical normal sample had one peak located at the DI value of 0.995 which indicated a diploid cell population (Fig 3A). A typical OLK sample showed multiple peaks in addition to the major diploid peak (e.g., DI = 0.798) (Fig 3D). A typical OSCC sample showed a peak pattern similar to that of an OLK sample (Fig 3G) often with more peaks beyond the DI of 2.3. In case there was only one diploid cell population, no more data processing was conducted (Fig 3B). Otherwise, data were further processed for extraction of the diploid and tetraploid cell populations, isolation of the aneuploidy cell population, and signal amplification. For a typical OLK sample and a typical OSCC sample, after the first cell population was extracted, the second peak and other small peaks became much more prominent (Fig 3E and 3H).

The major statistics of the diploid, tetraploid and aneuploid cell populations were then pooled together for data reconstruction. Along the x-axis of DI value, we defined finite number variables with a range of 0–8 and 16 intervals. As shown in boxplots, normal samples (n = 102), OLK samples (n = 82), and OSCC samples (n = 93) showed different patterns (Fig 3C, 3F, and 3I).

### Selection of SVM as the statistical model

**Fig 4:**
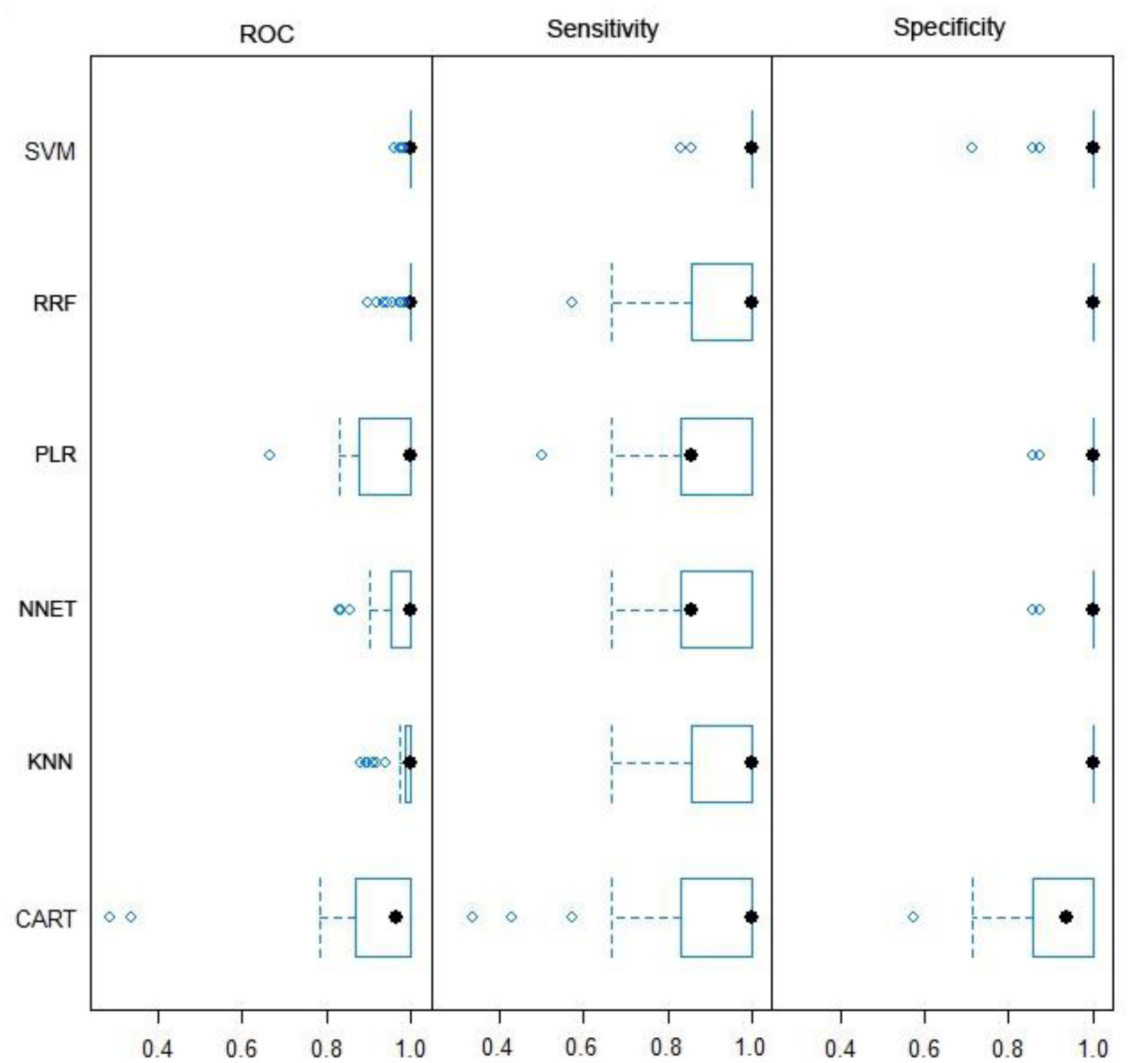
Assessment of statistical models. Six models (SVM, RRF, PLR, NNET, KNN, and CART) were tested for their performance using three parameters, ROC, sensitivity and specificity. Each model was trained on the training data and tested on the testing data. Each boxplot showed the distribution of these three parameters (R caret package http://cran.r-project.org/web/packages/caret/index.html).

Six statistical models were tested using data of normal and OSCC samples, which were pathologically distinct. Sensitivity, specificity and the area under the ROC curve were reported (Fig 4). Median sensitivity ranged between 0.83 and 1, with the SVM having the highest median sensitivity (S2 Table). Specificity was high for all six models indicating low false positive rates. Taking both the sensitivity and specificity into account, the area under the ROC curved provided a general fair assessment of the performance of a model. The median ranged between 0.91 and 1. As compared with the other five models, SVM performed the best, and thus was chosen as the statistical model for calculation of OCRI.

### Quantitative risk stratification of OLK patients

**Fig 5:**
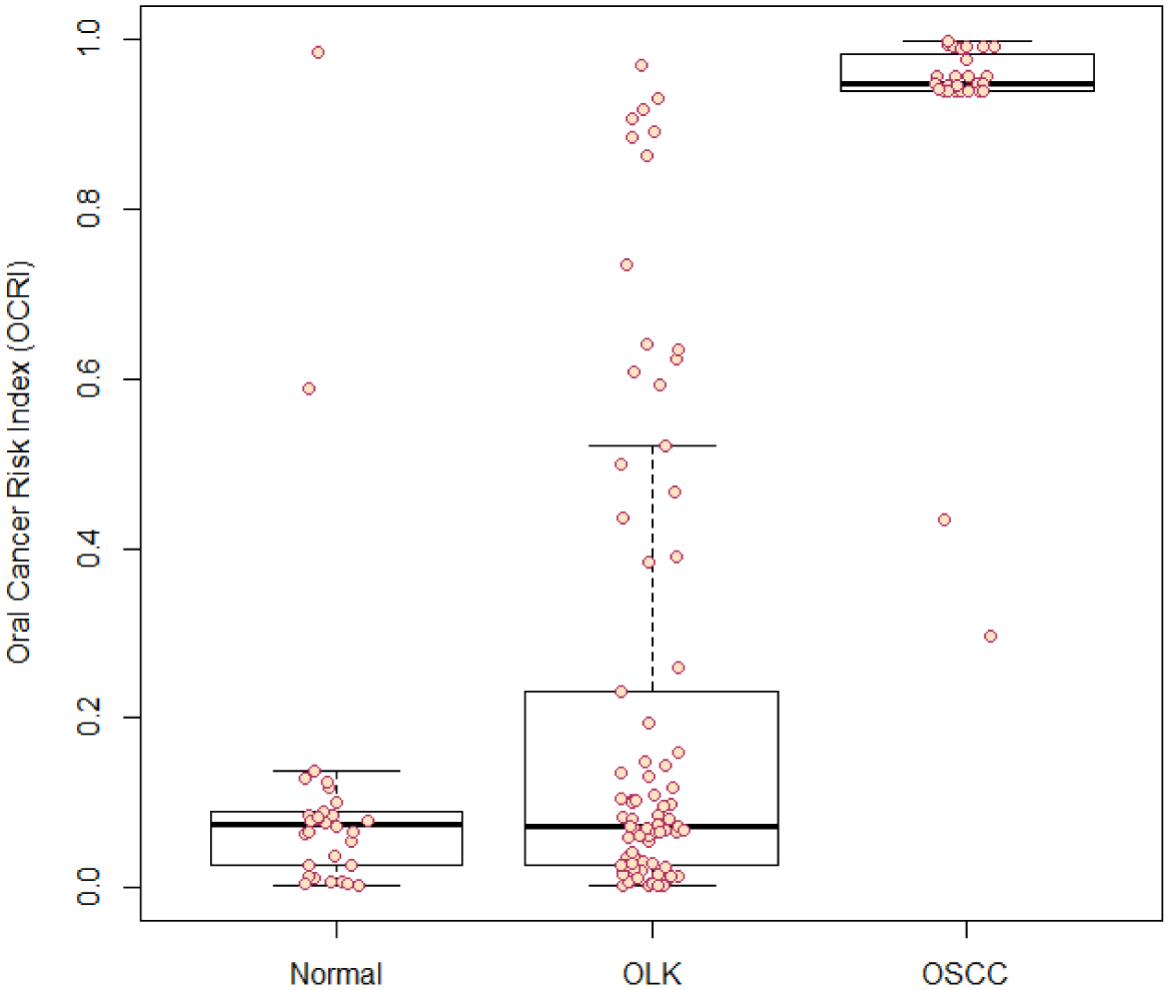
Oral Cancer Risk Index (OCRI) of normal subjects, OLK patients and OSCC patients. OCRI was calculated for each case with known pathology, and ranged between 0 and 1, where 0 indicates the lowest risk of OSCC and 1 indicates the highest risk of OSCC.

We further fine-tuned the SVM model with leave-one-out cross validation strategy and finalized on the key hyperparameters (cost C = 32 and hyperparameter sigma = 0.6456). The model was built with data of 70% cases (72 normal and 66 OSCC) and tested on data of the remaining cases. A sensitivity of 0.939, a specificity of 0.9444, and an area under ROC of 0.968 were reached. To calculate OCRI, we applied the model to data of a new sample, and let the model compute the probability that this sample was sampled from an OSCC population given the variables. OCRI was shown on the scale between 0 and 1 (y-axis). Data of 30 normal samples, 27 OSCC samples, and 82 OLK samples tested with OCRI were shown in the same scale (Fig 5).

The majority of normal samples were predicted with an OCRI<0.5 with two exceptions (0.98, 0.59). The majority of OSCC samples were predicted with an OCRI>0.5with two exceptions (0.30, 0.43). Consistent with the clinical nature of OLK, OCRI of the OLK samples spread across a wide range. Of the 82 OLK samples, 14 had an OCRI above 0.5 (S3 Table).

### Clinical follow-up of OLK patients

**Fig 6:**
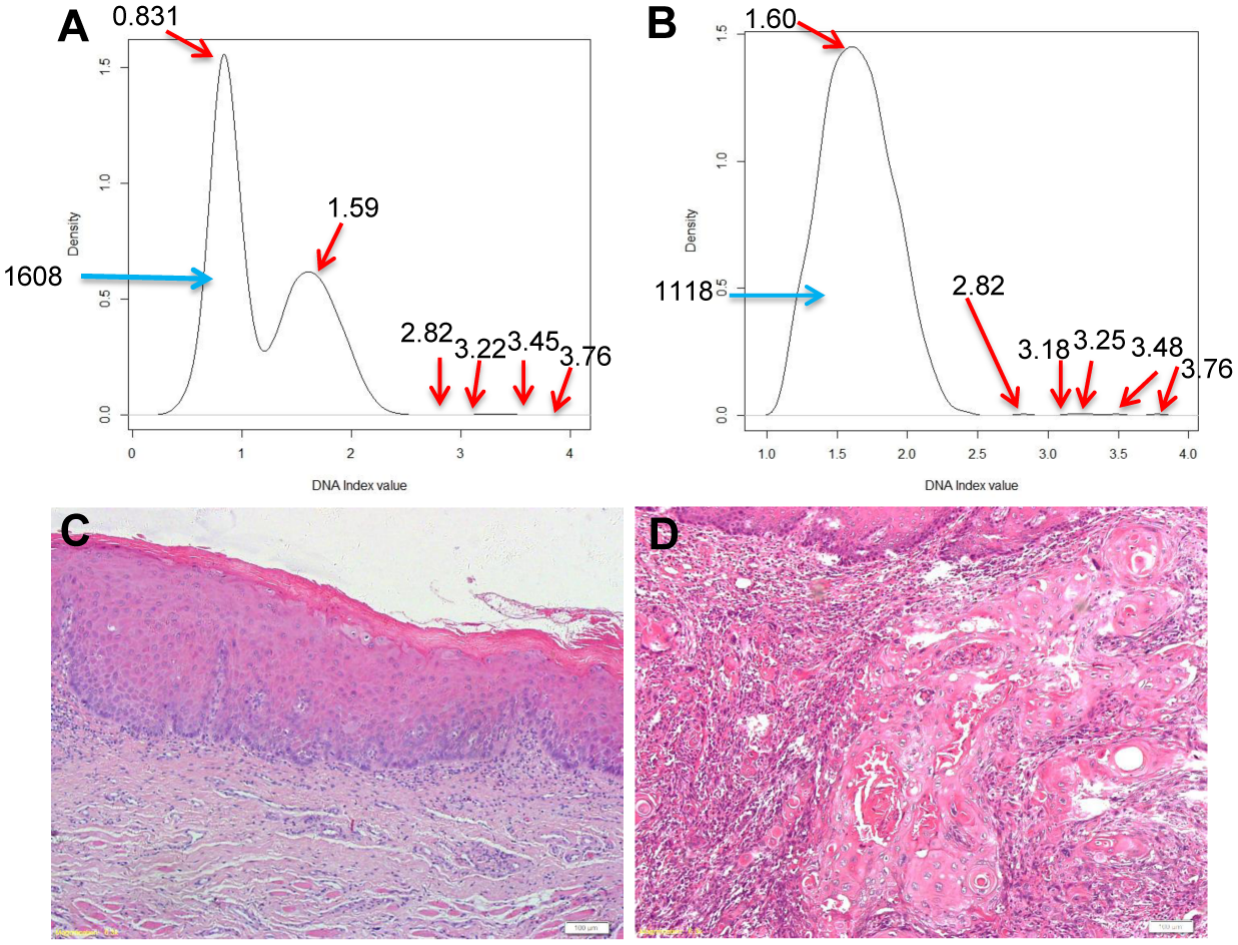
Application of EdTAR in clinical follow-up of one patient (Case 128141). Exfoliative cytology was performed in April 2008 and a density plot of DI data was generated (A).With EdTAR, positive signals were relatively amplified and an OCRI was calculated as 0.88 (B). Histopathology of biopsy showed mild dysplasia on H&E stained section, scale bar = 100 μm (C). A tumor was observed in August 2011 with a histopathological diagnosis of OSCC, scale bar = 100 μm (D).

The mean follow-up time for the OLK patients was 46 months. In one case (Case 128141, this individual has given written informed consent to publish this case details), the density plot of DI values obtained from initial exfoliative cytology showed multiple peaks in April 2008 (Fig 6A). The first two peaks represented diploid and tetraploid cell populations as the majority. Following data processing with EdTAR, the second peak became prominent after the first population was successfully extracted (Fig 6B). An OCRI was calculated as 0.88. Although biopsy histopathology reported mild dysplasia (Fig 6C), this patients was regularly followed up in our outpatient clinic. A tumor was observed in August 2011, and the histopathology confirmed the diagnosis of OSCC (Fig 6D).

## Discussion

In this study, we developed a statistical modeling method for quantitative risk stratification of OLK patients. Using a data transformation method (EdTAR) and a machine learning technique (SVM), we generated a quantitative index, OCRI, for assessment of cancer risk. This index is potentially useful for guiding clinical follow-up of OLK patients and improving cost-effectiveness. Further follow-up of our cases of OLK is expected to set a cutoff threshold of OCRI.

OLK, as a definite premalignant lesion of OSCC, is known to carry a cancer risk higher than normal subjects [14]. However, OLK may develop from multiple mechanisms some of which may not be associated with cancer risk at all. Visual inspection by clinicians with the aid of various tools tends to have a high rate of false positivity. As a well-established and widely used method for early detection of oral cancer, exfoliative cytology provides qualitative results. The major advantages are its being minimally invasive and inexpensive, and thus better acceptance by patients [25, 39]. In clinical setting, physicians have to reply on multiple tests during follow-up before the patient is definitely proved to be “positive”. Therefore there is a need of quantitative risk stratification of OLK. In this study, using DI values of exfoliative cytology we successfully developed EdTAR as a method for data transformation and reconstruction. This strategy overcomes the major problem in statistical analysis of exfoliative cytology data, which usually contain a big population of diploid cells, a smaller population of tetraploid cells, and a very small population of aneuploid cells. After EdTAR, the signal of aneuploid cell population is amplified. Reconstruction of data of three cell populations allows SVM for pattern recognition and calculation of OCRI. One of our OLK cases had a high OCRI and was found to develop OSCC 40 months later during follow-up.

Several approaches have been employed for quantitative stratification of cancer risk. Cancer risk index based on clinical risk factors, for example Harvard Cancer Risk Index [40], had only a modest discriminatory accuracy for several cancers. It is mainly used for the general population, but not in a tissue or cancer-specific manner for OLK patients. Recently there has been a tremendous enthusiam of using molecular markers for cancer risk stratification, such as mRNA expression data (using gene array, qRT-PCR)[41] and protein expression data (using immunohistochemical staining) [42]. This approach has been well developed for clinical use in breast cancer [43] and colon cancer [41]. However, performance of molecular markers is not much better than established risk factors. In one study [41], the four tested gene expression-based risk scores provide prognostic information but only contributed marginally to improving models based on established risk factors. It is believed that selection of prognostic gene lists and unclear biological significance of gene signatures contributed to this limitation. Combination with clinicopathological risk factors and inferring biologically relevant pathway deregulation scores have been proposed as potential solutions [44]. In oral cancer, a 29-gene predictive model showed marked improvements in terms of prediction accuracy over the models using previously known clinicopathological risk factors. The prediction error curves showed that Model 1 (only using microarray data) can markedly improve the prediction accuracy over Model 3 (clinical data and protein data). Model 2 (using microarray data, clinical data and protein data) was slightly better than Model 1, and both models have similar performance with 8% prediction error rate beyond 2 years of follow-up time [45]. Although this approach is promising, high cost, special expertise in sample analysis and data analysis, and high-quality sampling are obvious hurdles to overcome before it can be routinely used in clinical setting. It is also a challenge to develop a uniform gene list according to distinct gene lists generated by various studies [46].

As a laboratory assessment of cellular markers, exfoliative cytology remains a practical and reliable method for quantitative risk stratification of OSCC. It has been well established that DNA aneuploidy can predict malignancy prior to histopathology [47, 48]. As a non-invasive and inexpensive method, this approach has advantages over other methods: cellular morphology tends to be relatively stable than molecular markers. However, our method has its limitations as shown by the presence of 4 outliers, two cases of normal with high OCRI and two cases of OSCC with low OCRI. It is suggested that exfoliative cytology may be repeated if OCRI is high. In addition, exfoliative cytology needs a standardized procedure including brushing, Feulgen staining, and image capturing. Moreover, multiple parameters collected by exfoliative cytology other than DI value may be potentially used for model construction. One quantitative cytology study have showed statistically significant differences between aneuploid and diploid samples in nuclear perimeter, area, diameter, minimum and maximum Feret, etc [49]. With the wide use of NextGen sequencing in studies on OSCC [50, 51], we believe incorporation of these molecular markers may further improve the performance of the quantitative prediction model.

## Supporting Information

S1 Table A list of cytological features. (DOCX)

S2 Table Area under ROC, sensitivity and specificity of six statistical models (number of resamples = 50). (DOCX)

S3 Table The Oral Cancer Risk Index (OCRI) of OLK Patients. (DOCX)
