# Supplementary material for: Quantitative Risk Stratification of Oral Leukoplakia with Exfoliative Cytology
Source: PLoS One. 2015 May 15;10(5):e0126760. doi: 10.1371/journal.pone.0126760 (PMC4433206; doi:10.1371/journal.pone.0126760)
Supplement: S1 Table — (DOCX) [file pone.0126760.s001.docx]

S1 Table. Cytological Features List

| Cytological features |  |
| --- | --- |
| area | mean_radius |
| max_radius | var_radius |
| compactness | elongation |
| mean_intensity | var_intensity |
| DNA_index | DNA_amount |
| OD_maximum^a^ | OD_mean |
| OD_variance | OD_skewness |
| OD_kurtosis | entropy |
| homogeneity | energy |
| correlation | contrast |

^a^: OD: Optical Density
