# Supplementary material for: Quantitative Risk Stratification of Oral Leukoplakia with Exfoliative Cytology
Source: PLoS One. 2015 May 15;10(5):e0126760. doi: 10.1371/journal.pone.0126760 (PMC4433206; doi:10.1371/journal.pone.0126760)
Supplement: S2 Table — (DOCX) [file pone.0126760.s002.docx]

S2 Table. Area under ROC, sensitivity and specificity of six statistical models (number of resamples = 50)

| Parameter | Model | Min | 1^st^ quarter | Median | Mean | 3^rd^ quarter | Max | NA's |
| --- | --- | --- | --- | --- | --- | --- | --- | --- |
| Area under ROC | CART | 0.75 | 0.8571 | 0.9167 | 0.9098 | 0.9286 | 1 | 0 |
|  | SVM | 0.881 | 1 | 1 | 0.985 | 1 | 1 | 0 |
|  | PLR | 0.6905 | 0.8571 | 0.9388 | 0.9218 | 1 | 1 | 0 |
|  | NNET | 0.7857 | 0.898 | 0.9286 | 0.9323 | 1 | 1 | 0 |
|  | KNN | 0.7976 | 0.8776 | 0.9691 | 0.9373 | 1 | 1 | 0 |
|  | RRF | 0.8333 | 0.9388 | 1 | 0.9638 | 1 | 1 | 0 |
| Specificity | CART | 1 | 1 | 1 | 1 | 1 | 1 | 0 |
|  | SVM | 1 | 1 | 1 | 1 | 1 | 1 | 0 |
|  | PLR | 1 | 1 | 1 | 1 | 1 | 1 | 0 |
|  | NNET | 0.7143 | 1 | 1 | 0.9657 | 1 | 1 | 0 |
|  | KNN | 0.8571 | 1 | 1 | 0.9914 | 1 | 1 | 0 |
|  | RRF | 1 | 1 | 1 | 1 | 1 | 1 | 0 |
| Sensitivity | CART | 0.75 | 0.8571 | 0.9167 | 0.9098 | 0.9286 | 1 | 0 |
|  | SVM | 0.881 | 1 | 1 | 0.985 | 1 | 1 | 0 |
|  | PLR | 0.6905 | 0.8571 | 0.9388 | 0.9218 | 1 | 1 | 0 |
|  | NNET | 0.7857 | 0.898 | 0.9286 | 0.9323 | 1 | 1 | 0 |
|  | KNN | 0.7976 | 0.8776 | 0.9691 | 0.9373 | 1 | 1 | 0 |
|  | RRF | 0.8333 | 0.9388 | 1 | 0.9638 | 1 | 1 | 0 |
