# Supplementary material for: Quantitative Risk Stratification of Oral Leukoplakia with Exfoliative Cytology
Source: PLoS One. 2015 May 15;10(5):e0126760. doi: 10.1371/journal.pone.0126760 (PMC4433206; doi:10.1371/journal.pone.0126760)
Supplement: S3 Table — (DOCX) [file pone.0126760.s003.docx]

S3 Table. The Oral Cancer Risk Index (OCRI) of OLK Patients

| Case | Age | Sex | Site^a^ | Smoking | Drinking | Clinical classification | Pathological diagnosis | Exfoliative cytology | | Clinical follow-up | |
| --- | --- | --- | --- | --- | --- | --- | --- | --- | --- | --- | --- |
|  |  |  |  |  |  |  |  | Qualitative^b^ | OCRI | Duration (mo) | Outcome ^c^ |
| 128111 | 50 | F | T | N | N | Heterogeneous | mild dysplasia | Negative | 0.59 | 72 | No |
| 128113 | 55 | F | G | Y | N | Heterogeneous | mild dysplasia | Negative | 0.01 | 72 | No |
| 128114 | 57 | F | T | N | N | Homogeneous | moderate dysplasia | Negative | 0.26 | 71 | No |
| 128116 | 52 | M | T | Y | Y | Homogeneous | mild dysplasia | Atypical | 0.08 | 73 | No |
| 128119 | 57 | M | G | Y | N | Heterogeneous | no dysplasia | Negative | 0.07 | 71 | No |
| 128125 | 50 | F | T | N | N | Homogeneous | moderate dysplasia | Negative | 0.01 | 74 | No |
| 128126 | 64 | F | G | Y | N | Homogeneous | no dysplasia | Negative | 0.01 | 71 | No |
| 128128 | 47 | M | G | N | Y | Homogeneous | moderate dysplasia | Atypical | 0.05 | 71 | No |
| 128129 | 62 | F | G | N | N | Homogeneous | no dysplasia | Negative | 0.12 | 71 | No |
| 128131 | 68 | F | B | N | N | Homogeneous | mild dysplasia | Negative | 0.64 | 71 | No |
| 128132 | 52 | M | B | N | N | Homogeneous | mild dysplasia | Negative | 0.39 | - | Lost |
| 128133 | 46 | F | B | N | N | Homogeneous | no dysplasia | Negative | 0.01 | 71 | No |
| 128134 | 68 | F | G | N | N | Homogeneous | moderate dysplasia | Negative | 0.13 | 71 | Lost |
| 128136 | 46 | F | G | N | N | Homogeneous | no dysplasia | Negative | 0.06 | 71 | No |
| 128138 | 55 | M | T | Y | N | Homogeneous | moderate dysplasia | Negative | 0.07 | 71 | No |
| 128140 | 60 | F | T | N | N | Heterogeneous | moderate dysplasia | Negative | 0.11 | 71 | No |
| 128141 | 48 | F | T | N | N | Homogeneous | mild dysplasia | Positive | 0.88 | 40 | OSCC |
| 128145 | 65 | M | G | N | Y | Homogeneous | no dysplasia | Atypical | 0.16 | 71 | No |
| 128147 | 44 | F | B | N | N | Homogeneous | no dysplasia | Negative | 0.50 | 71 | No |
| 128149 | 52 | F | G | N | N | Homogeneous | moderate dysplasia | Negative | 0.00 | 71 | No |
| 128150 | 62 | F | T | N | N | Heterogeneous | no dysplasia | Atypical | 0.10 | 71 | No |
| 9449998 | 71 | F | T | N | N | Homogeneous | moderate dysplasia | Negative | 0.04 | 38 | No |
| 9449999 | 38 | F | G | N | N | Homogeneous | moderate dysplasia | Negative | 0.07 | 38 | No |
| 9450000 | 71 | F | G | N | N | Homogeneous | no dysplasia | Negative | 0.23 | 38 | No |
| 9558745 | 67 | M | B | N | N | Heterogeneous | moderate dysplasia | Negative | 0.07 | 42 | No |
| 9559194 | 75 | M | B | Y | N | Homogeneous | mild dysplasia | Atypical | 0.10 | 42 | No |
| 9559197 | 40 | F | B | N | N | Homogeneous | no dysplasia | Negative | 0.10 | 37 | No |
| 9559198 | 40 | F | G | N | N | Homogeneous | mild dysplasia | Negative | 0.03 | 36 | No |
| 9559200 | 40 | F | T | N | N | Homogeneous | mild dysplasia | Negative | 0.07 | 37 | No |
| 9559201 | 70 | M | T | Y | Y | Homogeneous | mild dysplasia | Atypical | 0.00 | 41 | No |
| 9559202 | 70 | M | T | Y | Y | Heterogeneous | moderate dysplasia | Positive | 0.00 | 41 | No |
| 9559231 | 48 | M | B | N | Y | Homogeneous | moderate dysplasia | Atypical | 0.08 | 41 | No |
| 9559232 | 76 | F | T | N | N | Homogeneous | moderate dysplasia | Positive | 0.08 | 41 | No |
| 9559233 | 59 | M | B | Y | Y | Homogeneous | mild dysplasia | Atypical | 0.04 | 41 | No |
| 9559234 | 70 | F | G | N | N | Homogeneous | moderate dysplasia | Negative | 0.10 | 41 | No |
| 9559235 | 70 | M | G | Y | N | Homogeneous | moderate dysplasia | Atypical | 0.10 | 41 | No |
| 9559237 | 58 | M | G | Y | Y | Homogeneous | mild dysplasia | Atypical | 0.06 | 41 | No |
| 9559238 | 58 | M | G | Y | Y | Homogeneous | mild dysplasia | Atypical | 0.02 | 41 | No |
| 9559239 | 60 | F | T | N | N | Homogeneous | moderate dysplasia | Negative | 0.02 | 41 | No |
| 9559240 | 68 | M | B | Y | N | Homogeneous | moderate dysplasia | Negative | 0.09 | 41 | No |
| 9559241 | 72 | F | T | N | N | Homogeneous | mild dysplasia | Atypical | 0.93 | 40 | No |
| 9559246 | 67 | F | B | N | N | Heterogeneous | no dysplasia | Negative | 0.91 | 40 | No |
| 9559247 | 67 | F | B | N | N | Homogeneous | no dysplasia | Negative | 0.97 | 40 | No |
| 9559248 | 59 | F | G | N | N | Homogeneous | mild dysplasia | Negative | 0.08 | 40 | No |
| 9559250 | 54 | F | G | N | N | Homogeneous | mild dysplasia | Negative | 0.02 | 41 | No |
| 9559261 | 52 | M | P | Y | Y | Heterogeneous | moderate dysplasia | Negative | 0.47 | 39 | No |
| 9559265 | 67 | M | B | Y | Y | Homogeneous | moderate dysplasia | Negative | 0.00 | 39 | N0 |
| 9559267 | 57 | M | G | N | N | Homogeneous | moderate dysplasia | Atypical | 0.92 | 40 | No |
| 9559272 | 67 | M | B | Y | Y | Heterogeneous | no dysplasia | Atypical | 0.38 | 36 | No |
| 9559275 | 52 | F | G | N | N | Homogeneous | mild dysplasia | Atypical | 0.01 | 39 | No |
| 9559277 | 54 | F | G | N | N | Homogeneous | no dysplasia | Atypical | 0.01 | 37 | No |
| 9559283 | 85 | M | P | N | Y | Homogeneous | no dysplasia | Negative | 0.86 | 37 | Death |
| 9559286 | 79 | F | G | Y | N | Homogeneous | moderate dysplasia | Atypical | 0.07 | 37 | No |
| 9559287 | 61 | F | G | Y | N | Homogeneous | mild dysplasia | Atypical | 0.02 | 37 | No |
| 9559288 | 54 | F | G | N | N | Homogeneous | moderate dysplasia | Atypical | 0.02 | 37 | No |
| 9559289 | 71 | F | G | N | N | Homogeneous | mild dysplasia | Negative | 0.03 | 37 | No |
| 9559290 | 25 | M | T | Y | N | Heterogeneous | moderate dysplasia | Atypical | 0.07 | - | Lost |
| 9559291 | 58 | F | B | N | N | Homogeneous | no dysplasia | Atypical | 0.03 | 38 | No |
| 9559292 | 65 | F | P | N | N | Homogeneous | no dysplasia | Negative | 0.04 | 37 | No |
| 9559295 | 52 | M | B | Y | N | Homogeneous | mild dysplasia | Negative | 0.01 | 37 | No |
| 9559296 | 68 | M | B | N | Y | Homogeneous | no dysplasia | Negative | 0.02 | 37 | No |
| 9559297 | 60 | F | T | N | N | Heterogeneous | moderate dysplasia | Atypical | 0.07 | 37 | No |
| 9559298 | 43 | M | T | Y | Y | Homogeneous | mild dysplasia | Negative | 0.02 | 37 | No |
| 9559398 | 60 | M | B | Y | N | Homogeneous | mild dysplasia | Atypical | 0.19 | - | Lost |
| 9559412 | 53 | F | T | N | N | Heterogeneous | mild dysplasia | Positive | 0.52 | 21 | No |
| 9559430 | 48 | F | T | N | N | Heterogeneous | moderate dysplasia | Atypical | 0.44 | 20 | No |
| 9559482 | 67 | F | B | N | N | Homogeneous | mild dysplasia | Negative | 0.15 | 32 | No |
| 9559486 | 55 | M | B | Y | N | Homogeneous | mild dysplasia | Negative | 0.06 | 31 | No |
| 9559488 | 39 | M | G | N | N | Homogeneous | mild dysplasia | Negative | 0.15 | 31 | No |
| 9559489 | 53 | F | T | N | N | Homogeneous | moderate dysplasia | Atypical | 0.62 | 31 | No |
| 9559490 | 60 | M | G | Y | Y | Heterogeneous | no dysplasia | Atypical | 0.03 | 31 | No |
| 9559491 | 47 | M | G | N | Y | Homogeneous | mild dysplasia | Atypical | 0.61 | - | Lost |
| 9559504 | 50 | M | B | Y | N | Homogeneous | mild dysplasia | Negative | 0.07 | 30 | No |
| 9559505 | 74 | M | G | N | N | Heterogeneous | mild dysplasia | Negative | 0.07 | 30 | No |
| 9559507 | 80 | F | B | N | N | Homogeneous | mild dysplasia | Negative | 0.14 | - | Lost |
| 9559542 | 29 | M | B | Y | N | Homogeneous | mild dysplasia | Negative | 0.10 | 35 | No |
| 9559546 | 65 | M | T | Y | Y | Homogeneous | mild dysplasia | Negative | 0.00 | 34 | No |
| 9559547 | 63 | M | G | Y | Y | Heterogeneous | no dysplasia | Atypical | 0.89 | 34 | No |
| 9559550 | 66 | F | G | N | N | Heterogeneous | moderate dysplasia | Negative | 0.07 | 34 | Death |
| 9559561 | 57 | M | B | Y | N | Homogeneous | moderate dysplasia | Negative | 0.63 | 35 | No |
| 9559565 | 44 | M | G | N | N | Homogeneous | no dysplasia | Atypical | 0.03 | 36 | No |
| 9559570 | 61 | F | G | N | N | Homogeneous | moderate dysplasia | Atypical | 0.73 | 36 | No |

^a^ B: Buccal, T: Tongue, G: Gingival, P: Palate

^b^ Negative, no aneupolid cells; Positive, definitive cellular evidence of epithelial dysplasia or carcinoma; Atypical, abnormal epithelial changes of uncertain diagnostic significance

^c^ OSCC, OSCC was diagnosed; No, No cancer was diagnosed; Lost; Lost in follow-up; Death: Death due to other diseases
